# Prognostic value of heart failure echocardiography index in HF patients with preserved, mid-ranged and reduced ejection fraction

**DOI:** 10.1186/s12872-020-01635-6

**Published:** 2020-07-29

**Authors:** Jian-Shu Chen, Ying Pei, Cai-e Li, Ning-yin Li, Tao Guo, Jing Yu

**Affiliations:** 1 Lanzhou University Second College of Clinical Medicine, Number 199 Donggang West Road, Chengguan District, Lanzhou, 730030 China; 2 Department of Cardiology, Second Hospital of Lanzhou University, No.82 Cui Ying Men, Cheng Guan District, Lanzhou, 730030 China

**Keywords:** Heart failure, Heart failure echocardiography index (HFEI), NT-proBNP, Prognosis

## Abstract

**Background:**

To investigate the clinical value of heart failure echocardiography index (HFEI) in evaluating the cardiac function and predicting the prognosis of patients with different types of heart failure (HF).

**Methods:**

Four hundred eighty-nine consecutively admitted HF patients were divided into three groups: HF with reduced ejection (HFrEF), HF with mid-range ejection fraction (HFmrEF), and HF with preserved ejection fraction (HFpEF). The baseline characteristics and ultrasound indexes were compared between the three groups. The correlation between HFEI and one-year risk of adverse events was compared by multivariate logistic regression. The clinical value of HFEI and plasma level of NT-proBNP in assessing the prognosis of patients with chronic heart failure (CHF) was analyzed by the receiver operating characteristic (ROC) curve.

**Results:**

HFEI in HFrEF was significantly higher than that in HFmrEF and HFpEF. Multivariate regression analysis indicated that HFEI and plasma level of NT-proBNP were independent risk factors for predicting the short-time prognosis of HF patients. The ROC curve indicated that the HFEI cutoff level of 3.5 and the plasma NT-proBNP level of 3000 pg/ml predicted a poor prognosis of CHF patients with a sensitivity of 64% and a specificity of 75% vs. 68 and 65%.

**Conclusion:**

HFEI can comprehensively evaluate the overall cardiac function of patients with various types of HF, and may prove to be an important index of assessing the prognosis of HF patients.

## Background

Chronic heart failure (CHF) represents the final common pathway for the development and progression of most cardiovascular diseases and the leading cause of death [1, 2]. Currently, the global prevalence of heart failure (HF) is about 2%, with an annual incidence of 1% [3]. In addition, with the global intensification of the aging process such as that in China, the number of HF patients continues to increase [4]. How to accurately diagnose and judge the prognosis in the early stage is the prerequisite and basis for effective treatment of HF patients.

Left ventricular ejection fraction (LVEF) and the left ventricular diastolic filling (LVDF) have long been used clinically to evaluate the LV systolic and diastolic functions respectively [5, 6]. However, LVEF or LVDF alone is not closely related to the clinical condition and cannot accurately reflect the overall cardiac function of HF patients. The HF echocardiography index (HFEI) is a combination of multiple parameters of echocardiography to evaluate the cardiac function in patients with CHF, which overcomes the subjective factors of New York Heart Association Classification and is more accurate and reliable than the single LVEF [7, 8].

The 2016 European Society of Cardiology (ESC) has updated its guidelines for HF. In addition to retaining HF with reduced ejection (HFrEF) and HF with preserved ejection fraction (HFpEF), a new type named HF with mid-range ejection fraction (HFmrEF) has been added [9]. Our study aims to investigate the value of HFEI in evaluating the cardiac function in patients with different types of HF and the clinical predictive value of HFEI for the prognosis of patients with CHF.

## Methods

### Study population

This study included 489 HF patients who were admitted to the Department of Cardiovascular Medicine of the Affiliated Hospital of Lanzhou University (Lanzhou, China) between January 2016 and December 2018. They included 268 males and 221 females, with a mean age of 64 ± 14 years. Inclusion criteria were as follows: (1) the diagnosis of heart failure is confirmed by two or more experienced cardiologists according to the 2016 ESC guidelines;(2) we can collect comprehensive clinical data; and (3) the one-year follow-up data of the patients were complete. The exclusion criteria were as follows: (1) patients with kidney disease, liver disease or severe liver and kidney dysfunction;(2) tumor, nervous system disease; All patients signed the informed consent forms before enrollment.

### Clinical data collection

The demographic characteristics and comorbidities of the patients were obtained on admission. All hematological specimens including blood routine, blood biochemistry or NT-proBNP were collected within 24 h after admission and tested in the central laboratory of the hospital. All clinical data were recorded by one physician and reviewed by another.

### Echocardiography

The GE Vivid 7 ultrasound system was used, with a probe frequency of 3.4–5.0 mHz. The measurement was performed by the same professional physician using the means of the results obtained from three consecutive cardiac cycles. Measurement indicators included: (1) the cardiac structure: LVEF, LV end diastolic diameter (LVEDD), interventricular septal thickness (IVST), LV posterior wall thickness (LVPWT), left atrial volume index (LAVi), diameter of left atrium (LAD) calculate relative ventricular wall thickness (RVWT) = 2 × LVPWTd/LVEDD, and LV mass index (LVMi) = LVM/body surface area; (2) cardiac function: peak velocity during early filing(E), late filling from atrial contraction (A), E/A ratio, left ventricular early diastolic flow propagation velocity (FPV), E/FPV, peak systolic mitral annular velocity(S′), E/E’S ratio, pulmonary arterial systolic pressure (PASP), early diastolic mitral flow deceleration time (DT), pulmonary venous flow (S peak systolic wave velocity and D peak diastolic wave velocity), and D/S ratio.

### HFEI

According to the related indexes of echocardiography, the total score was HFEI. The HFEI scoring criteria are as follows: (1) 35 ≤ PASP< 50 mmHg, 1 point, PASP≥50 mmHg, 2 point; (2) LV systolic function: 30% ≤ LVEF< 45% or regional ventricular wall motion abnormality, 1 point, LVEF< 30%, 2 point; (3) LV diastolic function: ①E/A < 0.5, DT > 220 ms, D/S < 1 or E/A = 1–2, DT = 150-220 ms, D/S ≥ 1, 1 point; ②E/A > 2, DT < 150 ms, D/S > 1 or restrictive change, 2 point; (4) atrioventricular remodeling: ①56 mm < LVEDD< 66 mm, IVST or LVPWT≥13 mm, LAD≥45 mm, 1 point; ②LVEDD≥66 mm or right ventricular dysfunction; (5) valvular regurgitation or valvular stenosis: moderate, 1 point, serious, 2 point (Table 1) [10, 11].

**Table 1:** Heart failure echocardiography index score criteria

	Indicators	Score
Left ventricular systolic function	30% ≤ LVEF< 45% or regional ventricular wall motion abnormality	1 point
	LVEF< 30%	2 point
Left ventricular diastolic function	E/A < 0.5, DT > 220 ms, D/S < 1 or E/A = 1–2, DT = 150-220 ms, D/S ≥ 1	1 point
	E/A > 2, DT < 150 ms, D/S > 1 or restrictive change	2 point
Pulmonary arterial systolic pressure	35 ≤ PASP< 50 mmHg	1 point
	PASP≥50 mmHg	2 point
Atrioventricular remodeling	56 mm < LVEDD< 66 mm, IVST or LVPWT≥13 mm, LAD≥45 mm	1 point
	LVEDD≥66 mm or right ventricular dysfunction	2 point
Valvular regurgitation or valvular stenosis	moderate	1 point
	serious	2 point

*DT* early diastolic mitral flow deceleration time, *D/S* pulmonary venous flow (S peak systolic wave velocity and D peak diastolic wave velocity), *E/A* peak velocity during early filing(E), late filling from atrial contraction (A), *IVST* interventricular septal thickness, *LVEF* left ventricular ejection fraction, *LVEDD* left ventricular end diastolic diameter, *PASP* pulmonary arterial systolic pressure, *LVPWT* left ventricular posterior wall thickness

### Follow-up evaluation and study end points

All patients were followed up on the outpatient basis, telephone interviews and readmission medical records, with a mean follow-up period of 1 year. End-point events observed in the study were all-cause death, cardiovascular death and hospitalization for HF exacerbation.

### Statistical analysis

All data were analyzed using SPSS 21.0 statistical software. Mean ± standard deviation (SD) was used for continuous variables which accord with the normal distribution and the homogeneity test of variance. The three groups of data were compared by the ANOVA test. Categorical variables were expressed as percentage and analyzed by the chi-square test. Univariate and multivariate logistic regression models were used to analyze the correlation between HFEI and the risk of one-year adverse events. The ROC curve was performed to evaluate the diagnostic performance of HFEI and NT-proBNP in patients with poor prognosis. A *p* value < 0.05 was pre-specified to indicate statistical significance.

## Results

### Comparison of the baseline characteristics

Four hundred and eighty-nine patients with HF were recruited and divided into three groups, including 170 in HFrEF, 171 in HFmrEF and 148 in HFpEF. There were no significant differences in age, diastolic blood pressure (DBP), blood urea nitrogen (BUN) and α-hydroxybutyrate dehydrogenase (α-HBDH) between the three groups of HF patients, while there were significant differences in HFEI and NT-proBNP between the three groups. The characteristics of the included patients are summarized in Table 2.

**Table 2:** Baseline characteristics of the study population

	HFrEF(*n* = 170)	HFmrEF(*n* = 171)	HFpEF(*n* = 148)	*p* value
Age (years)	62.20 ± 14.82	64.22 ± 13.18	67.02 ± 13.22	0.008*
Male, n(%)	98(57)	93(54)	79(53)	0.758
BMI (kg/m2)	25.25 ± 15.10	25.76 ± 16.53	25.36 ± 14.57	0.575
WHR	0.93 ± 0.06	0.92 ± 0.06	0.92 ± 0.08	0.575
SBP (mmHg)	135.19 ± 20.86	137.80 ± 25.71	137.42 ± 28.40	0.597
DBP (mmHg)	77.83 ± 12.36	80.64 ± 16.19	82.06 ± 16.14	0.043*
HR (beats/min)	78.75 ± 13.83	81.17 ± 16.02	78.88 ± 15.40	0.264
WBC(10^9/L)	6.35 ± 1.86	6.44 ± 1.85	6.42 ± 2.73	0.942
RBC(10^12/L)	4.68 ± 0.65	4.49 ± 0.68	4.56 ± 0.74	0.065
Hemoglobin (μmol/L)	143.83 ± 19.44	136.38 ± 20.17	138.58 ± 22.74	0.530
HCT	0.40 ± 0.09	0.41 ± 0.35	0.41 ± 0.08	0.821
ALT(U/L)	42.19 ± 56.06	34.44 ± 41.43	27.73 ± 22.84	0.120
AST(U/L)	33.91 ± 43.34	29.01 ± 20.40	25.92 ± 13.25	0.052
BUN (mmol/L)	8.15 ± 8.62	6.88 ± 2.93	6.69 ± 3.05	0.039*
CR (μmol/L)	91.47 ± 31.72	91.13 ± 38.32	86.54 ± 36.71	0.407
TC (mmol/L)	3.89 ± 1.06	3.82 ± 0.95	3.75 ± 1.08	0.512
HDL-C (mmol/L)	1.14 ± 0.33	1.15 ± 0.30	1.13 ± 0.33	0.881
LDL-C (mmol/L)	4.42 ± 2.62	2.26 ± 0.88	2.15 ± 0.89	0.401
UA (μmol/L)	394.90 ± 132.62	381.57 ± 130.29	364.08 ± 117.24	0.105
LDH(U/L)	209.76 ± 78.75	224.31 ± 100.24	209.18 ± 79.71	0.330
α-HBDH(U/L)	428.09 ± 263.19	243.54 ± 163.23	298.20 ± 160.83	0.015*
HCY (μmol/L)	20.79 ± 9.55	28.67 ± 38.31	26.07 ± 16.04	0.772
NT-proBNP (pg/ml)	6835.52 ± 6456.96	3628.96 ± 4997.18	1309.34 ± 1125.50	0.000*
Hypertension,n(%)	65(38)	89(52)	86(58)	0.006*
Diabetes,n(%)	33(19)	25(15)	38(26)	0.130
Atrial fibrillation,n(%)	15(9)	22(13)	21(14)	0.447

*ALT* alanine transaminase, *AST* aspartate aminotransferase, *α-HBDH* α-hydroxybutyrate dehydrogenase, *BUN* blood urea nitrogen, *BMI* body mass index, *CR* creatinine, *DBP* diastolic blood pressure, *HCT* hematocrit, *HR* heart rate, *HCY* homocysteine, *HDL-C* high-density lipoprotein cholesterol, *LDH* lactate dehydrogenase, *LDL-C* low-density lipoprotein cholesterol, *RBC* red blood cell, *SBP* systolic blood pressure, *TC* total cholesterol, *UA* uric acid, *WHR* waist hip ratio, *WBC* white blood cell

*p < 0.05 indicates a statistically significant differences among HFrEF, HFmrEF and HFpEF

### Comparison of the echocardiographic indexes

Analysis of the echocardiographic parameters in the three groups of patients showed that there were significant differences in the indicators that reflect the ventricular structure including LVEDD, LAD, LVMI and RWT, and in the indicators that reflect the ventricular function including E/FPV and E/E’S. No variances were found in the remaining indicators (Table 3).

**Table 3:** Echocardiographic indexes of the study population

	HFrEF(*n* = 170)	HFmrEF(*n* = 171)	HFpEF(*n* = 148)	*p* value
LVEF	30.78 ± 5.34	44.24 ± 3.87	61.45 ± 4.90	0.000
E/A	1.26 ± 0.71	1.14 ± 0.90	1.06 ± 0.81	0.389
E’L (cm/s)	7.65 ± 2.42	7.88 ± 2.64	7.74 ± 2.73	0.927
E/E’L	12.75 ± 7.61	12.48 ± 4.87	11.72 ± 5.27	0.396
E’S (cm/s)	4.81 ± 1.74	5.56 ± 2.15	5.79 ± 1.96	0.088
E/E’S	19.38 ± 11.27	17.15 ± 7.48	14.94 ± 5.61	0.037*
DT (ms)	182.01 ± 81.98	207.80 ± 79.58	221.62 ± 63.12	0.062
D/S	1.37 ± 0.87	1.49 ± 0.59	1.28 ± 0.43	0.019*
LVEDD (mm)	65.52 ± 8.93	58.23 ± 15.94	48.28 ± 9.01	0.000*
LAD (mm)	46.96 ± 11.04	44.09 ± 13.72	38.18 ± 9.29	0.000*
IVST (mm)	8.68 ± 2.20	8.92 ± 2.35	8.88 ± 2.28	0.062
PWT (mm)	9.15 ± 2.38	9.19 ± 2.14	9.71 ± 2.08	0.069
RWT (mm)	0.31 ± 0.08	0.42 ± 0.11	0.44 ± 0.11	0.000*
LVEDV (ml)	181.81 ± 63.51	119.32 ± 59.76	94.80 ± 38.02	0.000*
LVESV (ml)	120.12 ± 48.22	53.77 ± 40.87	37.24 ± 18.72	0.000*
LAVi	58.76 ± 7.58	50.59 ± 29.34	49.12 ± 18.15	0.135
IVRT (ms)	86.26 ± 28.45	87.46 ± 32.74	77.62 ± 23.93	0.093
Tei	0.64 ± 0.19	0.68 ± 0.20	0.62 ± 0.21	0.595
FPV (cm/s)	34.19 ± 13.01	45.33 ± 19.36	51.61 ± 21.44	0.000*
E/FPV	2.55 ± 0.94	2.09 ± 0.83	1.71 ± 0.67	0.000*
LVMi	167.43 ± 43.59	133.61 ± 37.51	115.49 ± 31.98	0.000*
HFEI	5.54 ± 1.20	4.12 ± 1.52	2.45 ± 1.16	0.001*

*DT* early diastolic mitral flow deceleration time, *D/S* pulmonary venous flow (S peak systolic wave velocity and D peak diastolic wave velocity), *E’L* maximum velocity in early diastole of mitral annulus- lateral wall, *E’S* maximum velocity in early diastole of mitral annulus- interventricular septum, *E/A* peak velocity during early filing(E), late filling from atrial contraction (A), *FPV* left ventricular early diastolic flow propagation velocity, *HFEI* heart failure echocardiography index, *IVRT* isovolumic relaxation time, *IVST* interventricular septal thickness, *LVEDV* left ventricular end diastolic volume, *LVESV* left ventricular end systolic volume, *LAD* diameter of left atrium, *LAVi* left atrial volume index, *LVMi* left ventricular mass index, *LVEDD* left ventricular end diastolic diameter, *LVEF* left ventricular ejection fraction, *PWT* posterior wall thickness, *RWT* relative wall thickness

*p < 0.05 indicates a statistically significant differences among HFrEF, HFmrEF and HFpEF

### Logistic regression analysis

Multiple logistic regression analysis was performed on variables that may affect the adverse prognosis of HF patients. It was found that NT-proBNP and HFEI were related to the risk of one-year adverse events. However, there was no correlation between LAVi, LVMi, α-HBDH and endpoint events (Table 4).

**Table 4:** Logistic regression analysis of one-year adverse event risk in patients with heart failure

Index	OR	95%CI	*p* value
Age	1.021	0.993–1.049	0.512
LAD	0.919	0.831–1.016	0.100
LVMi	1.002	0.986–1.081	0.759
α-HBDH(U/L)	1.001	0.999–1.004	0.282
HFEI	1.263	1.040–1.533	0.019
NT-proBNP (pg/ml)	1.000	0.966–1.025	0.010

*α-HBDH* α-hydroxybutyrate dehydrogenase, *CI* confidence intervals, *HFEI* heart failure echocardiography index, *LAD* left atrial volume index, *LVMi* left ventricular mass index

*p < 0.05 indicates a statistically significant differences

### The value of HFEI and NT-proBNP in predicting the one-year risk of adverse events in HF patients

**Fig. 1:**
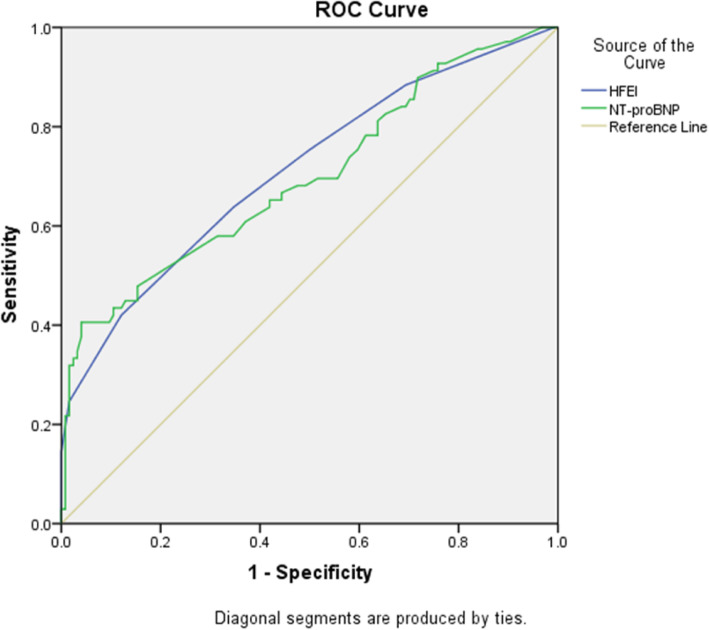
Receiver operating characteristic (ROC) curves of heart failure echocardiography index (HFEI) and NT-proBNP for predicting adverse events within 1 year in patients with heart failure. The maximum area under the ROC curve of heart failure echocardiography index and NT-proBNP was 0.712 and 0.698, respectively

The sensitivity and specificity of NT-proBNP and HFEI to predict the risk of one-year adverse events in patients with HF were analyzed by plotting the ROC curve, and the Jordon index was calculated. When NT-proBNP was 3000 pg/ml, the area under the ROC curve was 0.698(sensitivity 68%, specificity 65%). The greatest area under the ROC curve of HFEI was 0.712, and the optimal cut-off value was 3.5(sensitivity 64%, specificity 78%) (Fig. 1). According to the cutoff value of HFEI and NT-proBNP, HF patients were divided into the following four groups: group A: HFEI<3.5 and NT-proBNP<3000 pg/ml; group B: HFEI<3.5 and NT-proBNP>3000 pg/ml; group C: HFEI>3.5 and NT-proBNP<3000 pg/ml; group D: HFEI>3.5 and NT-proBNP>3000 pg/ml. The one-year risk of adverse events in HF patients between groups was analyzed using Partitions of χ2 method. The incidence of one-year adverse events in group A was lower than that in group C and D. Group D had a higher incidence of one-year adverse events than group D (Table 5).

**Table 5:** Partitions of χ2 method analysis of one-year adverse event risk in patients with heart failure

Group	One-year adverse events		All	χ2	*p*
	yes	no			
A	10	136	146	1.25	0.263>0.0083
B	11	90	101		
A	10	136	146	9.21	0.002<0.0083*
C	18	72	90		
A	10	136	146	18.16	0.000<0.0083*
D	38	114	152		
B	11	90	101	3.07	0.080>0.0083
C	18	72	90		
B	11	90	101	7.74	0.005<0.0083*
D	38	114	152		
C	18	72	90	0.8	0.373>0.0083
D	38	114	152		

A: HFEI<3.5 and NT-proBNP<3000 pg/ml; B: HFEI<3.5 and NT-proBNP>3000 pg/ml

C: HFEI>3.5 and NT-proBNP<3000 pg/ml; D: HFEI>3.5 and NT-proBNP>3000 pg/ml

*p < 0.0083 indicates a statistically significant differences

## Discussion

This observational study yielded the following results: (1) there were significant differences in HFEI between HFrEF, HFmrEF and HFpEF patients; (2) multivariate regression analysis indicated that HFEI and NT-proBNP were independent risk factors for the prognosis of one-year adverse events in HF patients; and (3) HFEI and NT-proBNP had a good value in predicting the short-term prognosis of HF patients.

Clinically, LVEF and LV filling indicators are generally used to evaluate the cardiac function in HF patients, but single indexes cannot reflect the overall cardiac function, especially in patients with HFpEF and HFrEF, in whom LVEF does not always reflect the severity of HF. HFEI is a comprehensive ultrasound index for evaluating the cardiac function by taking into full account the changes in LV systolic and diastolic function, valve regurgitation and atrioventricular remodeling [12, 13]. In addition, HFEI is not affected by the heart rate and ventricular geometry, and is highly correlated with the LV function indicators measured by cardiac catheterization, which can fully reflect the overall function of the heart. As the the present study has demonstrated significant differences in HFEI between the three types of HF patients, the evaluation of patients could be conducted objectively through the comprehensive echocardiographic parameters.

NT-proBNP is a cardiac neurohormone secreted by the ventricles, playing an important role in the diagnosis and prognosis of HF [14, 15]. However, studies have shown that plasma NT-proBNP level in patients with chronic kidney disease and cor pulmonale are also significantly increased, so NT-proBNP is not the specific diagnostic criterion for HF [16]. Several studies have shown a correlation between the multiple echocardiographic evaluation indicators (E/A and LVEF) included in HFEI and plasma NT-proBNP levels [8, 17]. Chen et al. suggested that HFEI could judge the degree of HF in HFmrEF patients and had a good correlation with NT-proBNP [18]. In this study, all the HF patients were followed up for 1 year to record the occurrence of adverse events and evaluate the prognosis. The result of multivariate logistic regression analysis also showed a significant correlation between NT-proBNP and short-term prognosis of HF patients. We also compared the value of HFEI and NT-proBNP in assessing short-term outcomes in HF patients. By plotting the ROC curve, it was found that the area under the curve for HFEI and NT-proBNP differed by 0.712 and 0.698, respectively. Therefore, NT-proBNP alone may not be sufficient to predict the risk of one-year adverse events in HF patients. Combination of a high plasma level of NT-proBNP and HFEI may improve the accuracy of prognostic prediction.

There are some shortcomings in this study. On the one hand, the study population was from a single center with a smaller sample size. On the other hand, the follow-up period was relatively short. Therefore, more studies with larger sample sizes and longer follow-up time are required to fully demonstrate the value of HFEI in the diagnosis and prognostic prediction of HF patients.

## Conclusion

In summary, the preliminary data obtained in this study suggest that HFEI could objectively predict the prognosis of patients with HF. HFEI and plasma NT-proBNP level are well correlated with the risk of adverse events, and combination of the two can improve the accuracy of prediction.

## Data Availability

Since some patients did not allow us to publish their medical records, datasets generated and/or analyzed in the current study are not publicly available, but can be obtained from the correspondence author on reasonable request.

## Abbreviations

CHF: Chronic heart failure
HF: Heart failure
LVEF: Left ventricular ejection fraction
LVDF: The left ventricular diastolic filling
HFEI: Heart failure echocardiography index
ESC: European society of cardiology
HFrEF: Heart failure with reduced ejection
HFpEF: Heart failure with preserved ejection fraction
HFmrEF: Heart failure with mid-range ejection fraction
LVEDD: Left ventricular end diastolic diameter
IVST: Interventricular septal thickness
LVPWT: Left ventricular posterior wall thickness
LAVi: Left atrial volume index
RVWT: Calculate relative ventricular wall thickness;
LVMi: Left ventricular mass index
E: Peak velocity during early filing
A: Late filling from atrial contraction
FPV: Left ventricular early diastolic flow propagation velocity
S’: Peak systolic mitral annular velocity
PASP: Pulmonary arterial systolic pressure
DT: Early diastolic mitral flow deceleration time
DBP: Diastolic blood pressure
BUN: Blood urea nitrogen
α-HBDH: α-Hydroxybutyrate dehydrogenase
